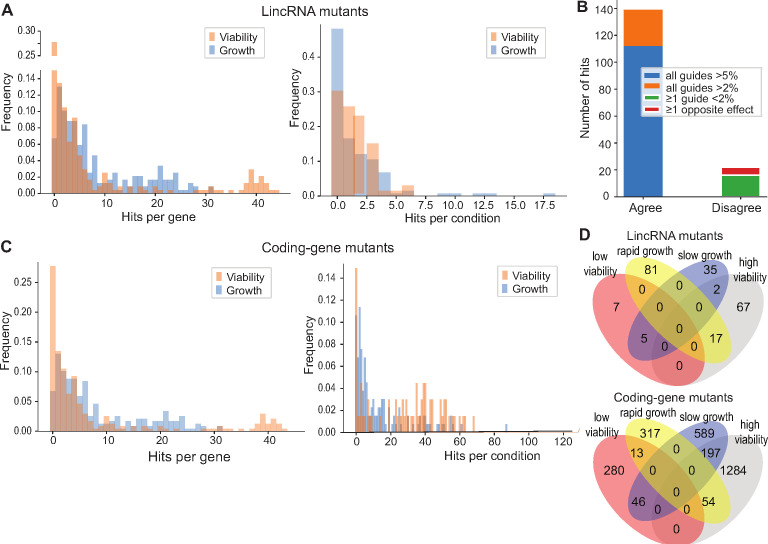# Correction: Functional profiling of long intergenic non-coding RNAs in fission yeast

**DOI:** 10.7554/eLife.77337

**Published:** 2022-01-27

**Authors:** Maria Rodriguez-Lopez, Shajahan Anver, Cristina Cotobal, Stephan Kamrad, Michal Malecki, Clara Correia-Melo, Mimoza Hoti, StJohn Townsend, Samuel Marguerat, Sheng Kai Pong, Mary Y Wu, Luis Montemayor, Michael Howell, Markus Ralser, Jürg Bähler

**Keywords:** *S. pombe*

Rodriguez-Lopez M, Anver S, Cotobal C, Kamrad S, Malecki M, Correia-Melo C, Hoti M, Townsend S, Marguerat S, Pong SK, Wu MY, Montemayor L, Howell M, Ralser M, Bähler J. 2022. Functional profiling of long intergenic non-coding RNAs in fission yeast. eLife **11**:e76000. doi: 10.7554/eLife.76000

Published 5 January 2022

We discovered that the wrong data were used for the left graph in Figure 4A, resulting in the left graphs of Figure 4A and 4C to show the same data. This error has been corrected by replacing the plot in Figure 4A with the correct plot showing the data for lincRNA mutants. We have also improved the right graph of Figure 4C by cutting the X axis to better represent the data range. The article has been corrected accordingly, and these corrections do not in any way change our conclusions.

The corrected Figure 4 is shown here:

**Figure 1:**
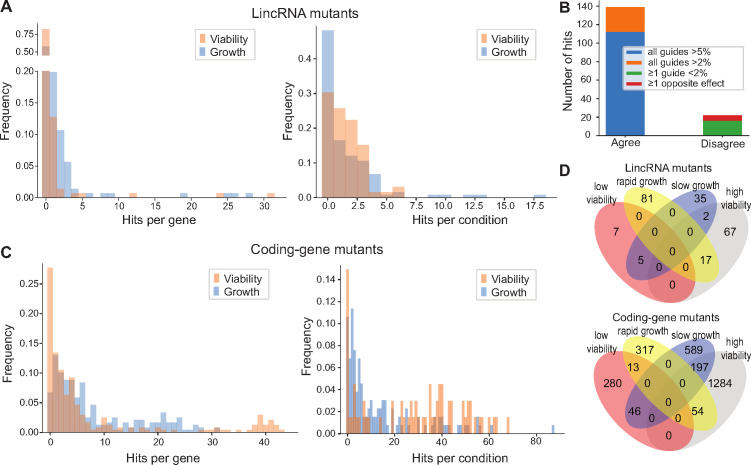


The originally published Figure 4 is shown below for reference:

**Figure 2:**